# Counter-flow elutriation of clinical peripheral blood mononuclear cell concentrates for the production of dendritic and T cell therapies

**DOI:** 10.1186/s12967-014-0241-y

**Published:** 2014-09-17

**Authors:** David F Stroncek, Vicki Fellowes, Chauha Pham, Hanh Khuu, Daniel H Fowler, Lauren V Wood, Marianna Sabatino

**Affiliations:** Department of Transfusion Medicine, Cell Processing Section, Clinical Center, National Institutes of Health (NIH), 10 Center Drive-MSC-1184, Building 10, Room 3C720, Bethesda, MD USA; Experimental Transplantation and Immunology Branch, National Cancer Institute (NCI), NIH, Bethesda, Maryland USA; Vaccine Branch, NCI, NIH, Bethesda, Maryland USA

**Keywords:** Cellular therapy, Cell processing, Counter-flow elutriation, Dendritic cells, Adoptive cell therapy

## Abstract

**Introduction:**

Peripheral blood mononuclear cells (PBMC) concentrates collected by apheresis are frequently used as starting material for cellular therapies, but the cell of interest must often be isolated prior to initiating manufacturing.

**Study design and methods:**

The results of enriching 59 clinical PBMC concentrates for monocytes or lymphocytes from patients with solid tumors or multiple myeloma using a commercial closed system semi-automated counter-flow elutriation instrument (Elutra, Terumo BCT) were evaluated for quality and consistency. Elutriated monocytes (n = 35) were used to manufacture autologous dendritic cells and elutriated lymphocytes (n = 24) were used manufacture autologous T cell therapies. Elutriated monocytes with >10% neutrophils were subjected to density gradient sedimentation to reduce neutrophil contamination and elutriated lymphocytes to RBC lysis.

**Results:**

Elutriation separated the PBMC concentrates into 5 fractions. Almost all of the lymphocytes, platelets and red cells were found in fractions 1 and 2; in contrast, most of the monocytes, 88.6 ± 43.0%, and neutrophils, 74.8 ± 64.3%, were in fraction 5. In addition, elutriation of 6 PBMCs resulted in relatively large quantities of monocytes in fractions 1 or 2. These 6 PBMCs contained greater quantities of monocytes than the other 53 PBMCs. Among fraction 5 isolates 38 of 59 contained >10% neutrophils. High neutrophil content of fraction 5 was associated with greater quantities of neutrophils in the PBMC concentrate. Following density gradient separation the neutrophil counts fell to 3.6 ± 3.4% (all products contained <10% neutrophils). Following red cell lysis of the elutriated lymphocyte fraction the lymphocyte recovery was 86.7 ± 24.0% and 34.3 ± 37.4% of red blood cells remained.

**Conclusions:**

Elutriation was consistent and effective for isolating monocytes and lymphocytes from PBMC concentrates for manufacturing clinical cell therapies, but further processing is often required.

## Introduction

Peripheral blood mononuclear cells (PBMC) concentrates collected by apheresis are rich in lymphocytes, monocytes and NK cells and they are a common source of starting material for the manufacture of cell and gene therapies. PBMC concentrates are used as a source of monocytes for the manufacture of dendritic cells (DCs), lymphocytes for cytokine stimulated and genetically engineered T cell therapies and natural killer (NK) cells for autologous and allogeneic immunotherapy. They also contain red blood cells (RBCs), platelets and neutrophils and in order to consistently produce high quality products, it is often necessary to isolate monocytes, lymphocytes or NK cells from PBMC concentrates.

When PBMCs are used as a source of autologous monocytes to manufacture DCs for cancer or antiviral immunotherapy the manufacturing process requires that monocytes are enriched from the PBMC concentrates. The monocytes are then incubated with agents such as GM-CSF and IL-4 for 3 to 7 days to differentiate them into immature DCs and with further culture on to mature DCs [1,2]. A variety of methods can be used to isolate monocytes from PBMCs, but the manufacturing of DCs for clinical therapy requires large scale isolation and the maintenance of the sterility of the cells. One Good Manufacturing Practices (GMP) method to isolate monocytes from PBMCs is immunomagnetic separation using CD14 monoclonal antibodies conjugated to magnetic particles in the CliniMACS system which yields monocytes of more than 90% purity with recoveries of approximately 65 to 80% [3–6]. The antibodies and sterile disposable kits used in this process are costly, but despite this limitation many laboratories use these them to isolate monocytes [3,4] to manufacture clinical cell therapies.

Another option for the isolation of monocytes from PBMC concentrates is continuous counter-flow elutriation. Elutriation uses centrifugal force against a continuously increasing fluid flow to separate cells based on size and to a lesser extent density. An automated continuous counter-flow elutriation instrument is available that has been used primarily to isolate monocytes from PBMC concentrates, the Elutra system. This instrument makes use of a closed system sterile plastic disposable kit that allows for the sterile processing of the cells [7–11]. The PBMC concentrate is loaded into the elutriator and cells are collected in 5 fractions. Typically, lymphocytes and red blood cells are found in earlier factions and neutrophils and monocytes in the later fractions. The cost of isolating monocytes using elutriation is less than that of using antibodies and magnetic beads. We have been using elutriation to enrich monocytes from PBMC concentrates to manufacture DCs for clinical vaccine studies.

PBMC concentrates are also used as a source of allogeneic lymphocytes from hematopoietic stem cell (HSC) transplant donors to treat patients with disease relapse following HSC transplantation; furthermore autologous PBMCs are used as a source of lymphocytes for adoptive immunotherapy of cancer. Aliquots of unprocessed PBMC concentrates can often be used for donor lymphocyte infusions, but for adoptive immunotherapy, lymphocytes generally must be free from contaminating RBCs and neutrophils. Immunomagnetic selection with CD3, CD4 or CD8 monoclonal antibodies conjugated to magnetic particles can also be used to select or deplete T cells or specific subsets of T cells [12–15]. We have been using counter-flow elutriation to isolate autologous lymphocytes for adoptive immune therapy. Since the elutriated fractions that contain lymphocytes also contain RBCs, we treat the elutriated lymphocytes to reduce RBC contamination prior to beginning lymphocyte culture.

While elutriation has been used to enrich for and separate monocytes [10,16] and lymphocytes [17] for clinical applications, little information is available on the consistency and quality of monocyte and lymphocyte concentrates obtained from PBMC concentrates collected for autologous clinical therapy. We summarized our experience using an automated instrument to elutriate 59 PBMC concentrates, which were processed to enrich for either monocytes to manufacture clinical DC products or lymphocytes to manufacture T cell products for clinical immunotherapy.

## Methods

### Study design

Subjects were enrolled in IRB-approved protocols that made use of mature DCs to treat adults with prostate or other advanced cancers or rapamycin-generated Th1/Tc1 lymphocytes [18,19] to treat patients with multiple myeloma. After obtaining informed consent, PBMCs were collected from subjects using a blood cell separator. The PBMC concentrates were elutriated with a semi-automatic counter-flow elutriator. All products elutriated from patients enrolled in three clinical protocols from May 2010 through December 2013 were studied. When monocytes isolated by elutriation were being used to manufacture DCs, if the proportion of neutrophils in the monocytes obtained from the elutriator was greater than 10%, the product was further enriched for monocytes using density gradient separation. When lymphocytes isolated by elutriation were used to manufacture clinical cell therapies, contaminating red blood cells were lysed using ammonium chloride.

### PBMC concentrate collection

PBMC concentrates were collected using a blood cell separator (COBE Spectra Apheresis System, Terumo BCT, Lakewood, Colorado, USA) by processing 8 to 12 liters of whole blood.

### Elutriation

The PBMC concentrates were subject to elutriation using a semi-automatic counter-flow elutriation instrument (Elutra Cell Separation System, version 1.1, Terumo BCT) using a user defined profile. Our procedure required that the quantity of white blood cells loaded into the Elutra be at least 5×10^9^ cells but not more than 20×10^9^ cells. The program for the collection of monocytes collects cells in 5 fractions. The chamber rotation speed was maintained at 2400 RPMs for fractions 1 through 4 and the media flow rate was maintained at 60 ml/min for fraction 1, 120 ml/min for fraction 2, 122 ml/min for fraction 3 and 124 ml/min for fraction 4. Fraction 5 was the cells remaining in the chamber and they were collected with the rotor turned off.

### Density gradient separation

The leukocytes were pelleted by centrifugation (350×g) for 10 minutes and resuspended in Hank’s Balance Salt Solution (HBSS) (Lonza, Walkersville, MD) at a concentration of 1-2×10^8^ cells per mL. Density gradient separation was performed by placing 40 mL of leukocyte suspension in a 50 ml conical tube and underlying cells with 10 mL of Ficoll/Hypaque [BioWhittaker Lymphocyte Separation Medium, Ficoll and sodium diatrizoate (Hypaque), density 1.077, Lonza]. After centrifugation at 835×g for 25 minutes the mononuclear cells were removed, washed (675×g, for 10 minutes) and resuspended in HBSS.

### RBC lysis

After pelleting the cells, they were incubated for 7 to 10 minutes with ACK Lysing Buffer (Lonza, Walkersville, MD) and then washed and resuspended in 0.9% saline (B.Braun Medical Inc., Irvine, CA) with 0.3% trisodium citrate (TriCitrasol anticoagulant sodium citrate concentrate 46.7%, Citra Labs, Braintree, MA).

### Cell counts

Blood counts were measured using automated hematology analyzer (Cell-Dyn 3700).

### Statistically analysis

The values shown are mean ± 1 standard deviation unless otherwise indicated. Groups were compared using t-tests (Microsoft Excel, Microsoft Inc., Redmond, WA).

## Results

### Subjects studied

A total of 59 autologous elutriated PBMC concentrates were studied which were collected from 51 subjects: PBSCs were collected from 6 subjects twice and 1 subject three times. Elutriated monocytes from 35 PBMC concentrates were used to manufacture autologous DCs for immunotherapy of cancer and elutriated lymphocytes from 24 PBMC concentrates were used to manufacture rapamycin-generated Th1/Tc1 lymphocytes cells for adoptive cell therapy following autologous transplantation of patients with multiple myeloma. 35 subjects were male and 16 were female and 42 were White, 7 were African American, 1 was mixed and for 1 the race was not known. The median age of the subjects was 59.3 years and ranged from 35.7 to 77.3 years.

### Composition of elutriated fractions

Analysis of all 59 elutriated PBMC concentrates found that the majority of leukocytes were found in fractions 1, 2 and 5 (Table 1). Almost all of the lymphocytes were found in fractions 1 and 2, 45.8 ± 24.8% and 35.1 ± 16.9% respectively, and almost all of the monocytes, 88.6 ± 43.0%, and neutrophils, 74.8 ± 64.3%, were in fraction 5. The lymphocyte rich fractions 1 and 2 contained almost all of the RBCs and platelets.

**Table 1:** **Composition of 59 clinical PBMC concentrates loaded into the counter-flow elutriation instrument and the 5 recovered fractions**

	**WBCs (×10** ^**9**^ **)**	**RBCs (** **×10** ^**9**^ **)**	**Platelets (** **×10** ^**9**^ **)**	**Neutrophils (** **×10** ^**9**^ **)**	**Lymphs (** **×10** ^**9**^ **)**	**Monos (** **×10** ^**9**^ **)**
**Loaded**	15.4 ± 4.2	199.9 ± 109.0	469.5 ± 194.2	1.81 ± 1.90	9.97 ± 3.28	3.14 ± 1.88
**Fraction 1**	5.04 ± 3.27	190.3 ± 123.9	349.8 ± 241.3	>0.100	4.68 ± 3.00	0.180 ± 0.316
**Fraction 2**	3.82 ± 2.07	58.4 ± 87.9	10.3 ± 60.4	>0.100	3.45 ± 1.94	0.299 ± 0.425
**Fraction 3**	0.38 ± 0.34	3.14 ± 1.90	>0.100	>0.100	0.26 ± 0.27	0.11 ± 0.21
**Fraction 4**	0.29 ± 0.30	0.95 ± 1.11	0.13 ± 0.52	>0.100	0.12 ± 0.11	0.17 ± 0.27
**Fraction 5**	4.16 ± 2.07	0.63 ± 1.46	0.16 ± 0.26	1.31 ± 1.77	0.16 ± 0.17	2.57 ± 1.13

The values shown are mean ± 1 standard deviation.

**Figure 1:**
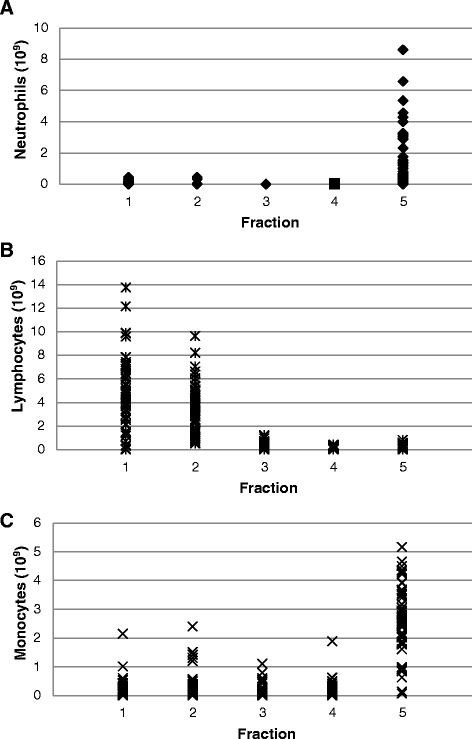
**Separation of neutrophils, lymphocytes and monocytes by counter-flow elutriation.** The results of the separation of neutrophils (panel **A**), lymphocytes (panel **B**) and monocytes (panel **C**) from 59 PBMC concentrates into fractions 1, 2, 3, 4 and 5) are shown. Each symbol represents one fraction from each of the 59 PBMC concentrates. Values are expressed as 10^9^ cells.

**Figure 2:**
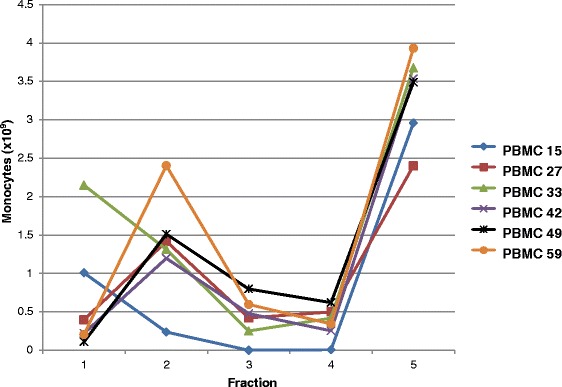
**Quantity of monocytes in each of the 5 fractions elutriated from 6 PBMC concentrates with large quantities of monocytes in fractions 1 or 2.** Results of elutriating 6 PBMC concentrates 15 (♦), 27(■), 33(▲), 42(×), 49(*), and 59(●) are shown. Values are expressed as 10^9^ cells.

The separation of neutrophils and lymphocytes was more consistent than that of monocytes. For all 59 elutriated PBMC concentrates fractions 1 through 4 contained less than 1×10^9^ neutrophils (Figure 1A) and fractions 4 and 5 contained less than 1×10^9^ lymphocytes (Figure 1A) but fraction 3 from one PBMC concentrate contained more than 1×10^9^ lymphocytes. However, fractions 1 and/or 2 from 6 PBMC concentrates contained more than 1×10^9^ monocytes (Figure 1C). For these 6 PBMC concentrates the quantities of monocytes in each of the 5 fractions is shown in Figure 2. A comparison of these 6 PBMC concentrates with the 53 concentrates with less than 1×10^9^ monocytes in fractions 1 through 4 found that the 6 PBMCs contained greater quantities of total white blood cells (18.3 ± 0.88×10^9^ versus 15.0 ± 4.28×10^9^, p < 0.035) and monocytes (5.07 ± 2.55×10^9^ versus 2.92 ± 1.65×10^9^, p = 0.0073) but similar quantities of neutrophils, lymphocytes, platelets and RBCs.

Our clinical DC manufacturing protocols use fraction 5 as a source of starting material for manufacturing DCs. While the majority of leukocytes in the 5 fraction were monocytes (68.1 ± 22.4%), a large quantity of neutrophils were present (24.8 ± 23.1%). Our protocols require that monocytes used to manufacture DCs contain 10% or less neutrophils. Among the 59 PBMC concentrates in this study, 35 were used to manufacture DCs and only 12 of the 35 fraction 5′s (34%) contained 10% or less neutrophils. The 23 fraction 5′s with >10% neutrophils were subjected to density gradient separation over ficoll to separate the monocytes and neutrophils. Following ficoll density gradient separation all 23 products contained less than 10% neutrophils and few monocytes were lost (Table 2).

**Table 2:** **Results of density gradient separation from elutriate fraction 5 (n = 23)**

	**WBCs (×** **10** ^**9**^ **)**	**Neutrophils (** **×10** ^**9**^ **)**	**Lymphs (** **×10** ^**9**^ **)**	**Monos (** **×10** ^**9**^ **)**	**Neutophils (%)**	**Lymphs (%)**	**Monos (%)**
**Pre-Ficoll**	4.33 ± 2.33	1.85 ± 2.08	0.16 ± 0.13	2.27 ± 0.89	35.0 ± 19.8	3.6 ± .2.9	59.4 ± 19.2
**Post-Ficoll**	2.47 ± 0.95	0.10 ± 0.12	0.08 ± 0.09	2.24 ± 0.84	3.61 ± 3.37	3.61 ± 7.34	91.0 ± 4.06

The values shown are mean ± 1 standard deviation.

When all 59 PBMC concentrates were considered, a comparison of the quantities of the various types of cells among the 38 concentrates that yielded fraction 5′s with >10% neutrophils and the 21 whose fraction 5′s yielded ≤10% revealed that PBMC concentrates yielding >10% neutrophils in fraction 5 contained significantly more neutrophils (Table 3). There were no other significant differences among the two groups of products.

**Table 3:** **Comparison of elutriated PBMC concentrates that yielded elutriated fraction 5s with >10% neutrophils and those with ≤10% neutrophils**

		**Composition of PBMC products loaded**					
**Neutrophils in Fraction 5**	**N**	**WBCs (** **×10** ^**9**^ **)**	**RBCs (** **×10** ^**9**^ **)**	**Platelets (** **×10** ^**9**^ **)**	**Neutrophil (** **×10** ^**9**^ **)**	**Lymphs (** **×10** ^**9**^ **)**	**Monos (** **×10** ^**9**^ **)**
**>10%**	38	15.6 ± 4.0	200.4 ± 120.1	459.6 ± 204.6	2.30 ± 1.91*	9.79 ± 3.26	3.18 ± 1.84
**≤10%**	21	14.9 ± 4.5	199.9 ± 85.2	488.9 ± 172.7	0.91 ± 1.51	10.30 ± 3.30	3.06 ± 1.93

The values shown are mean ± 1 standard deviation.

*p <0.01.

We also used elutriation to isolate lymphocytes from PBMC products that are used to manufacture Th1/Tc1 cells. Fraction 2 or fraction 2 plus fraction 1 are used. Both fractions 1 and 2 contain large quantities of lymphocytes with few other leukocytes, but fraction 2 contains fewer RBCs and platelets and is the preferred faction for our manufacturing protocol. However, in order to obtain sufficient quantities of lymphocytes both fractions are often used. Among the products studied, 24 were used to manufacture rapamycin-generated Th1/Tc1 lymphocytes cells; for 7 products only fraction 2 was used for further manufacture and for 17 products, fractions 1 and 2 were used. All 24 elutriated lymphocytes used for further manufacturing were subjected to ammonium chloride RBC lysis which was effective at removing RBCs with little loss of lymphocytes. Recovery of WBCs following RBC lysis was 86.7 ± 24.0% and 34.3 ± 32.4% of the RBCs remained.

### Comparison of elutriated products among subjects

Among the 59 elutriated PBMC concentrates, 28 were from men with stage 0 prostate cancer whose only measure of disease was a rising Prostate Specific Antigen (PSA) (biochemical recurrence) and the other 31 products were from patients with either multiple myeloma or various advanced solid cancers. In general, the health of the patients with prostate cancer was better than that of the other patients. To determine if the condition of people donating PBMC concentrates influenced the effectiveness of the counter-flow elutriation process we compared the composition of the cells loaded into the elutriation instrument and the elutriated fractions among these two groups. Fraction 5 from patients with multiple myeloma and advanced cancers had significantly more monocytes and lymphocytes but similar number of neutrophils (Table 4). This is likely a result of loading the elutriator with slightly more WBCs and monocytes from patients with multiple myeloma and advanced cancer. The proportion of leukocytes in fraction 5 that were neutrophils were similar for patients with prostate cancer and multiple myeloma/advanced cancer (25.9 ± 22.1% versus 23.9 ± 23.9%, p = 0.75 respectively) as were the proportion of monocytes (68.6 ± 22.0% versus 67.5 ± 22.8%; p = 0.85). Among 28 elutriated PBMC concentrates from men with prostate cancer, 2 contained more than 1×10^9^ monocytes in fractions 1 or 2 and among the 31 from patients with multiple myeloma or advanced cancer 4 contained more than 1×10^9^ monocytes.

**Table 4:** **Comparison of PBMC cellular content relative to elutriated fraction 5 from patients with early stage prostate cancer and multiple myeloma and other advanced stage cancers**

	**n**	**WBCs (** **×10** ^**9**^ **)**	**RBCs (** **×10** ^**9**^ **)**	**Platelets (** **×10** ^**9**^ **)**	**Neutrophils (×** **10** ^**9**^ **)**	**Lymphs (×** **10** ^**9**^ **)**	**Monos (** **×10** ^**9**^ **)**
**Loaded**							
**Prostate Cancer**	28	14.1 ± 4.5	212.6 ± 130.2	524.9 ± 196.5	1.56 ± 1.69	9.66 ± 3.39	2.58 ± 1.49
Multiple Myeloma*	31	16.6 ± 3.6^#^	188.4 ± 83.7	421.3 ± 178.7^#^	2.03 ± 2.04	10.3 ± 3.15	3.64 ± 2.04^#^
**Fraction 5**							
**Prostate Cancer**	28	3.59 ± 2.18	1.02 ± 1.88	0.24 ± 0.29	1.29 ± 1.99	0.086 ± 0.09	2.11 ± 0.81
Multiple Myeloma*	31	4.66 ± 1.81^$^	0.28 ± 0.76	0.09 ± 0.20^$^	1.32 ± 1.54	0.22 ± 0.20^$^	2.98 ± 1.22^$^

The values shown are mean ± 1 standard deviation.

*Includes patients with multiple myeloma and cancers other than prostate cancer.

^#^p < 0.05 for comparison of the quantity of cells loaded in the elutriator among patients with prostate cancer and multiple myeloma and other advanced cancers.

^$^p < 0.05 for comparison of the quantity of cells in fraction 5 among patients with prostate cancer and multiple myeloma and other advanced cancers.

## Discussion

We used an automated counter-flow elutriation instrument to enrich monocytes from PBMC concentrates to manufacture clinical DC products and clinical rapamycin-generated Th1/Tc1 lymphocytes for autologous adoptive immunotherapy. When PBMC concentrates from patients with prostate cancer, multiple myeloma and other advanced malignancies were processed with the elutriation instrument, lymphocytes were easily, consistently and cost effectively separated from monocytes. Almost all monocytes were found in fraction 5 and almost all lymphocytes were present in fractions 1 and 2. However, almost all neutrophils were also found in fraction 5 with monocytes and almost all RBCs were in fractions 1 and 2 with lymphocytes. These results are similar to those of other reports which evaluated the same instrument to isolate monocytes from small numbers of PBMC concentrates collected from healthy subjects [7,9,11,20–23], pooled whole blood buffy coat products [8,24] and PBMCs from up to 24 patients with cancer [10,11,16].

We found that the separation of monocytes was less consistent than that of lymphocytes and neutrophils. For 10% of the PBMC concentrates elutriation resulted in the distribution of relatively large quantities of monocytes into fractions 1 or 2. This was not a failure to separate monocytes since significant quantities of monocytes were also found in fraction 5. The fact that PBMCs concentrates with large quantities of monocytes in fractions 1 or 2 contained larger quantities of white blood cells and monocytes suggest that loading too many monocytes into the elutriation instrument may result in suboptimal monocyte separation. Our elutriation protocol limited the number of white blood cells loaded to ≤ 20×10^9^ cells but there were no upper limits on the quantity of monocytes loaded.

While counter-flow elutriation was consistent and effective at separating lymphocytes and monocytes, the elutriated cells of interest commonly require further processing before they could be placed into culture. In order to ensure consistent manufacturing of DCs from monocytes, our manufacturing protocols require that the monocytes used for starting material contain less than or equal to 10% neutrophils. We found that only about one-third of the products met these criteria, but the quantity of neutrophils among the monocytes could be reduced greatly by density gradient separation with little loss of monocytes. We investigated factors that might influence the neutrophil content of the elutriated monocytes and found that the portion of neutrophils in the monocyte rich fraction 5 was dependent on the quantity of neutrophils loaded into elutriator at the onset of the process; this result is similar to the findings of Chen et al. [10]. While we elected to deplete neutrophils from some elutriated monocytes, others have reported that the presence of as much as 20% to 30% neutrophils in elutriated monocytes used to manufacture DCs does not affect the quality of the final mature DC product [24]. If we would have used >30% as criteria for neutrophil reduction, 19 (32%) of the fraction 5 isolates would, still have required further density gradient separation.

When the counter-flow elutriator was used to enrich lymphocytes from PBMC concentrates, we used the lymphocyte rich fractions 1 or 2 or only fraction 2, depending on the quantity of lymphocyte required. However, RBCs were found in fractions 1 and 2 with fraction 1 containing approximately 3 times more RBCs than fraction 2. In order to obtain RBC-depleted lymphocytes, the desired fractions were treated with ACK lyse solution to lyse contaminating RBCs. This process reduced the number of RBCs approximately 3-fold with only a small reduction of lymphocyte quantity. In most cases the isolated lymphocytes were cryopreserved prior to manufacturing the rapamycin-generated Th1/Tc1 lymphocytes. This freeze/thaw cycle resulted in further lysis and depletion of RBCs (data not shown).

While both density gradient separation and RBC lysis were effective at increasing the purity of the elutriated monocytes and lymphocytes, they required the use of additional reagents and staff time. In addition, both density gradient separation and lysis procedures were semi-open manual processes which increased the risk of microbial contamination of the final product. Ideally, it would be best to avoid further processing of monocytes and lymphocytes isolated by counter-flow elutriation by collecting PBMC concentrates that have very low quantities of neutrophils and RBCs, but this was not always possible. The quantities of neutrophils and RBCs contaminating PBSC concentrates collected by apheresis is dependent on several factors including the donors neutrophil count, RBC characteristics, duration of the apheresis procedure and characteristics of the blood cell separator. All blood cell separators collect some neutrophils and RBCs with the lymphocytes and monocytes [25,26] and the quantities of neutrophils and RBCs collected increases as the volume of blood processed during the apheresis procedure increases. The DC manufacturing protocols require relatively large quantities of monocytes and hence a relatively large volume of blood was processed and large quantities of neutrophils are collected. The quantities of neutrophils collected with lymphocytes and monocytes are dependent to some degree on the blood cell separator. The Fenwal CS3000 was more efficient at collecting mononuclear cells, but it is no longer available [25]. The blood cell separator we used in this study is only partially automated; a new more highly automated blood cell separator is becoming available which may be able to collect PBMC concentrates with a greater proportion of monocytes [26] and hence would require a shorter collection duration resulting in PBMC concentrates with fewer contaminating and neutrophils.

## Conclusions

Elutriation is effective for isolating monocytes from PBMC concentrates for DC manufacture, but the elutriated monocyte fraction often required further processing to reduce the neutrophil content. Elutriation was also effective at obtaining lymphocytes from PBSC concentrates, but all lymphocytes fractions contained RBCs and an RBC lysis step was also required prior to initiating cultures. The separation of monocytes was less consistent that of lymphocytes and inefficient separation was associated with loading the elutriator with large quantities of monocytes.

## Abbreviations

DC: Dendritic cell
HBSS: Hank’s balance salt solution
HSC: Hematopoietic stem cell
NK: Natural killer
Lymphs: Lymphocytes
PBMC: Peripheral blood mononuclear cells
Monos: Monocytes
RBCs: Red blood cells
